# Special Issue “Ligament/Tendon and Cartilage Tissue Engineering and Reconstruction”

**DOI:** 10.3390/ijms27073181

**Published:** 2026-03-31

**Authors:** Clemens Gögele, Gundula Schulze-Tanzil

**Affiliations:** Institute of Anatomy and Cell Biology, Paracelsus Medical University, 90419 Nuremberg, Germany; clemens.goegele@pmu.ac.at

## 1. Introduction

Connective tissues such as ligaments, tendons, and cartilage have numerous shared tissue properties and are characterized by no or only poor blood supply and a low proportion of cellular components (~<10%), which are incorporated into a highly specialized extracellular matrix (ECM), which is largely composed of collagen [1]. Collagen type I is the dominant protein in ligaments and tendon tissue. The parallel-running dense fiber bundles of collagen type I resist tensile forces, while collagen type III provides structural flexibility and is formed during ligament/tendon tissue repair. Proteoglycans such as decorin and biglycan regulate collagen fibrillogenesis and maintain fiber spacing [2]. Collagen type 5 (COL5A1) is important for the structural integration of ligaments and therefore influence their mechanical properties [3]. Glycoproteins like tenascin C and fibronectin support cell adhesion, mechanotransduction, and tissue resilience [4,5]. Chondromodulin (Cnmd), described in detail in this Special Issue, is a glycoprotein in cartilage that is highly implicated in the inhibition of angiogenesis and cartilage homeostasis. Interestingly, it shows a high level of homology with tenomodulin in ligaments and tendons (contribution 8). In contrast to ligament and tendon ECM, the articular cartilage ECM is rich in collagen type 2, which forms a fibrillary network that provides tensile strength. Collagen type 5 can be found in both cartilage and in ligament/tendons responsible for controlling fibril diameter [6]. The major proteoglycan, aggrecan, binds large amounts of water through its glycosaminoglycan chains, giving cartilage its typical resistance to compression. Some of these tissue characteristics explain the poor self-healing abilities of ligament/tendon and cartilage. Accordingly, biofabrication is a promising strategy for supporting tissue repair and reconstruction. Using novel biomimetic materials serving as chondro-/tenogenic cell carriers presents an innovative culturing approach (e.g., using biomaterials like poly-ε-caprolactone (PCL) or silk, with different manufacturing techniques, for appropriate topology and cell sources that could support the regeneration of ligament/tendon tissues). The cells recruited for tissue engineering, the tailored release of bioactive factors, targeted cell lineage differentiation, predisposition for injury, and markers for tracing healing success altogether remain significant research interests.

In this Special Issue, original research papers and reviews on the following topics have been published:

1. Embroidered silk fibroin scaffolds provide a reproducible architecture with tunable porosity and mechanical properties, supporting ligament fibroblast colonization and ligament-specific ECM expression. Hence, silk scaffolds represent promising candidates for anterior cruciate ligament (ACL) biofabrication (contribution 1).
2. The study in question reveals pronounced location-dependent variations in the ECM composition of porcine knee articular cartilage and shows significant differences in cartilage thickness, proteoglycan content, and key ECM components such as collagen type 2, aggrecan, decorin, and glycoproteins like cartilage oligomeric protein and fibrillin-1 between the medial and lateral femoral knee cartilage compartments. The special heterogeneity in cartilage ECM composition, likely shaped by local mechanical loading, must be considered in cartilage tissue engineering and repair strategies (contribution 2).
3. Juvenile chondrocytes isolated from polydactyly-affected digits and the iliac apophysis can be successfully harvested and up-scaled in a GMP-compatible Quantum^®^ bioreactor while retaining their key chondrogenic characteristics. Manufacturing scalable numbers of allogeneic chondrocytes from juvenile donors is feasible and could enable cost-effective chondrocyte implantation therapies for large cartilage defects (contribution 3).
4. This systematic review and meta-analysis reveals that the thymin/thymin (TT) genotype (where thymin is on both alleles) of the collagen type 5A1 (COL5A1) rs13946 variant is associated with greater susceptibility to ACL injuries in a recessive genetic model, particularly in the Caucasian population (contribution 4).
5. The application of Light-Emitting Diode (LED) photo-biomodulation via irradiation at 630 nm and 880 nm wavelengths improves Achilles tendon healing in mice by enhancing fiber organization, increasing tenocyte density, and reducing fibrosis. Treatment with LED photo-biomodulation promotes a shift in macrophage polarization toward the anti-inflammatory M2 phenotype and is therefore a promising therapeutic approach for tendon injuries (contribution 5).
6. The original research paper “Effect of collagen coating and fiber profile on tenocyte growth on braided poly-ε-caprolactone (PCL) scaffolds for tendon and ligament regeneration” demonstrates that noncircular (but with “snowflake” like cross-sections) PCL fibers, especially when coated with collagen, significantly enhance tenocyte adhesion, proliferation, and alignment compared to circular fibers. Tailoring the cross-section and surface functionalization are key factors in improving the cellular performance of braided scaffolds for tendon/ligament tissue engineering (contribution 6).
7. The transplantation of amniotic epithelial stem cells allows modulation of tendon healing via time-dependent downregulation of the expression of neural markers (nerve growth factor [NGF], calcitonin gene-related peptide [CGRP], neurofilament [NF]-200, and neuropeptide [NPY]), indicating a controlled neural response in the early healing of ovine tendons. This property is correlated with improved tendon ECM remodeling and fiber organization. The specific patterns of neural marker expression can serve as predictors for favorable versus impaired tendon regeneration (contribution 7).
8. Cnmd is a cartilage-specific ECM component and plays a central role in cartilage homeostasis, promoting chondrocyte proliferation, inhibiting angiogenesis, and contributing to bone repair. The downregulation of Cnmd is associated with osteoarthritis progression. This suggests that Cnmd is a promising therapeutic and diagnostic target in cartilage and joint diseases (contribution 8).

**Figure 1:**
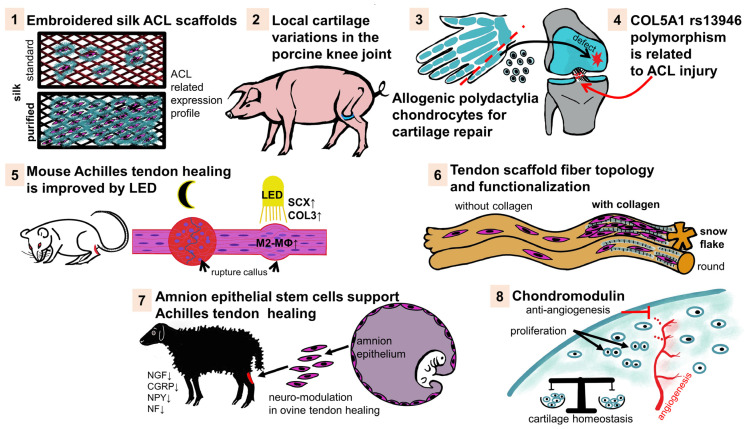
Tendon and ligament tissue engineering and reconstruction. 1: enhanced growth of cruciate ligament fibroblasts (pink) on purified versus standard embroidered silk scaffolds. 3: dotted red line: resection of a surplus 6th finger: chondrocyte isolation and transplantation in a joint cartilage defect (black arrow). 5: the arrow indicates the lesser mature (left, under normal light/darkness) and more mature callus (after exposition to LED) in the healing tendon. 7: the arrows show that amnion epithelial stem cells were isolated and transplanted into an ovine Achilles tendon defect. 8: the arrows indicate chondrocyte proliferation, red lines: inhibition of angiogenesis. ACL: anterior cruciate ligament, COL3: collagen type 3, COL5A1: collagen type 5 alpha1 chain, LED: light-emitting diode, M2-Mφ: M2 polarized anti-inflammatory macrophages, SCX: scleraxis. Images were created by G. Schulze-Tanzil using krita (version 4.4.7). **1–8**: Special Issue contributions.

Eight articles have been published, all of which are summarized in the scheme below (Figure 1) and Table 1.

## 2. Future Perspectives in the Field of Ligament/Tendon and Cartilage Tissue Engineering and Reconstruction

To translate the collective findings of the above-mentioned studies into clinical reality as rapidly as possible, we propose a pragmatic roadmap that integrates materials with tailored topology and functionalization addressing the specific cell biology requirements. The following rigorous approaches should be considered: Engineer scaffolds with tailored microarchitectures and surface functionalization (noncircular fiber profiles and collagen/peptide coatings) to direct ligamentocyte/tenocyte alignment and cartilage-specific ECM deposition. Couple different materials with targeted immunomodolatory mediators or potent perinatal/allogeneic cell types to support the tissue regeneration process. Products should be manufactured in a GMP-compatible pipeline as early as possible. Incorporate non-cellular adjuncts such as calibrated photo-biomodulation to boost early repair responses and synchronize cellular activity in order to achieve controlled ECM remodeling and inhibition of fibrosis/adhesion or scarring in tendon. Using spatial ECM mapping and biomechanic analysis can help generate defect-location-matched implants, and this approach might be superior to the application of one-size-fits-all constructs. Molecular and genetic biomarkers (neural/ECM markers, e.g., Cnmd or COL5A1 variants) could be integrated into the preclinical workflows to stratify patients and estimate individual healing outcomes. Finally, validate combinations in large-animal studies with harmonized outcome measures, scale up manufacturing and quality controls, and proceed to staged, well-powered clinical trials to demonstrate safety, efficacy, and reproducibility.

## Figures and Tables

**Table 1:** Contributions in this Special Issue.

Article Type	Topic	Main Message	Contributions
Original research	ACL/material	Embroidered silk fibroin scaffolds are promising for ACL reconstruction	**1**
Original research	Cartilage	The importance of ECM variations in joint cartilage depends on joint localization and zones	**2**
Original research	Cartilage	An allogeneic cartilage defect therapy with juvenile chondrocytes was developed	**3**
Systematic Review/ Meta-analysis	ACL	COL5A1 rs13946 polymorphisms correlate with the risk of ACL injury	**4**
Original research	Tendon	LED photo-biomodulation improved Achilles tendon repair	**5**
Original research	Tendon/material	PCL fiber cross-section and surface functionalization with collagen are essential to improve tenocyte growth	**6**
Original research	Tendon	The neuro-modulatory activity of amniotic epithelial stem cells casts them as key predictive biomarkers for tendon healing	**7**
Narrative review	Cartilage	Cnmd should be further analyzed as a potential therapeutic and diagnostic target.	**8**

## Abbreviations

The following abbreviations are used in this manuscript:

ACL	Anterior cruciate ligament
Cnmd	Chondromodulin
COL	Collagen
ECM	Extracellular Matrix
LED	Light-Emitting Diode
PCL	poly-ε-caprolactone
